# The Effect of Intestinal Parasitic Infection on the Clinical Outcome of Malaria in Coinfected Children in Cameroon

**DOI:** 10.1371/journal.pntd.0004673

**Published:** 2016-04-29

**Authors:** Tebit E. Kwenti, Franklin A. Nkume, Ajime T. Tanjeko, Tayong D. B. Kwenti

**Affiliations:** 1 Department of Medical Laboratory Sciences, University of Buea, Buea, Cameroon; 2 Diagnostic Laboratory, Regional Hospital Buea, Buea, South West Region, Cameroon; 3 Department of Microbiology and Parasitology, University of Buea, Buea, Cameroon; Queensland Institute for Medical Research, AUSTRALIA

## Abstract

**Background:**

The interaction between intestinal parasites and malaria is still not clear. Data in published literature are conflicting. We studied the effect of intestinal parasitic infection (IPI) on the clinical outcome of malaria in coinfected children.

**Methods:**

In a cross sectional study performed between October 2014 and September 2015, children infected with malaria, as demonstrated by the presence of asexual parasites in Giemsa stained blood films, were enrolled. Stool samples were obtained from participants and subjected to the formol-ether concentration technique for the detection of intestinal parasites. The Complete blood count was performed using an automated haematology analyser (Mindray, BC-2800). The risk ratio, Pearson’s chi-square and the student T test were all performed as part of the statistical analyses. Statistical significance was set at p < 0.05.

**Results:**

In all, 405 children successfully took part in the study. The children were between 1 week and 120 months of age (mean ± SD = 41.5 ± 33.5). Coinfection with intestinal parasites was observed in 11.6%. The rate of severe malaria (SM) attack in this study was 10.9%. SM was not observed to be associated with age (p = 0.377) or gender (p = 0.387), meanwhile coinfection with intestinal parasites was associated with age (p = 0.003). Among SM cases, IPI prevalence was higher in children with mild (WHO group 3) severe malaria (p = 0.027). Overall, IPI was not observed to be associated with SM (p = 0.656) or malaria parasite density (p = 0.185) or haemoglobin concentration (p = 0.205). The main clinical features of SM observed were hyperpyrexia (68.2%), severe malarial anaemia (61.4%), and multiple convulsion (52.3%).

**Conclusion:**

IPI was not observed to be associated with the severity of malaria, the malaria parasite density, and the haemoglobin concentration in coinfected children in Cameroon. The clinical outcome of malaria in children coinfected with intestinal parasites may depend on the geographical setting after all.

## Author Summary

Coinfection with malaria and intestinal parasites are common in Sub-Saharan Africa, particularly in impoverished and poor sanitary settings. The interaction between intestinal parasites and malaria in coinfected children is still not clear. Some published papers suggest intestinal parasites, especially *Ascaris lumbricoides*, may attenuate the severity of malaria in the presence of coinfection. In this cross-sectional study, we evaluated the effect of intestinal parasitic infection on the severity of malaria, malaria parasite density and the haemoglobin concentration in children coinfected with malaria and intestinal parasites in Cameroon. We did not observe any significant association between intestinal parasitic infection and severe malaria or malaria parasite density or haemoglobin concentration. Stratification of severe malaria according to the degree of severity revealed a significant association with intestinal parasitic infection, in which prevalence of intestinal parasites was higher in children with mild severe malaria. Analyzing the different species of intestinal parasite did not yield any significant association either. These findings are contrary to many research publication on the subject. Several factors could have contributed to our observation, including the regular deworming campaign organized by the Cameroon Ministry of Public health, accounting for the lower prevalence of intestinal parasitic infection, and also the geographical setting.

## Introduction

Malaria and intestinal parasitic infections (IPIs) are parasitic diseases that are highly endemic in Sub-Saharan Africa, especially in impoverished and poor sanitary settings. In 2013, there were 198 million cases and 584000 deaths as a result of malaria worldwide [1]. The majority of deaths due to malaria are reported in Sub-Saharan Africa and occur mostly in children below 15years [2]. Five species of *Plasmodium* are known to cause malaria in humans including *P*. *falciparum*, *P*. *ovale*, *P*. *vivax*, *P*. *malariae*, and *P*. *knowlesi*. Among them, *P*. *falciparum* is the most virulent species responsible for a majority of cases and almost all malaria associated deaths [3,4]. Like malaria, the prevalence of IPIs is also higher in developing countries, reaching up to 95% in some settings in Sub-Saharan Africa. IPIs are caused by either helminths, protozoa or both. Because of the overlapping distribution of malaria and IPIs, coinfection between malaria and IPI are therefore common in Sub-Saharan Africa [5]. The interaction between malaria and the different intestinal parasites whenever coinfection is present is poorly understood.

Infection with intestinal parasites especially helminths is strongly suspected to influence the incidence, parasite density and the clinical outcome of malaria in endemic areas. Earlier studies have reported increased susceptibility to malaria [6–8], increased malaria gametocyte carriage [9], decreased haemoglobin concentration [10] and increased risk for clinical and severe malaria [11–13] in helminth coinfected individuals. Conversely, other studies have reported that helminth infections may protect from malaria, or related clinical outcomes by suppressing acute clinical manifestations [14,15], parasite density [16,17] or severe complication such as cerebral malaria [18], circulatory collapse [19], renal failure and jaundice [20]. Yet in other studies, no significant association have been observed [7,21]. The variations in the results obtained could be attributed to the different helminth species, different transmission settings, and the complex nature of the immune responses to malaria parasites or the altered immune response due to helminth co-infections. In addition, variations in the study design or methodology, case definition or malaria severity status, stage or intensity of species of helminths or *Plasmodium* and other confounding factors could equally contribute to the varying results observed in these studies [22–24].

In Cameroon, like most Sub-Saharan African countries, coinfection between malaria and intestinal parasites is common in children [25–28]. Across the country, the prevalence of coinfection with malaria and intestinal parasites varies from 11.9% to 34.7% [25–29] and school-age children are the most affected. IPI may exacerbate the severity of malaria in these children. To the best of our knowledge, no study has been performed in Cameroon to determine the interaction between malaria and intestinal parasitic infection. This study was therefore designed to give an insight on the effect of IPIs on the clinical outcome of malaria (such as severe malaria and parasite density) in coinfected children in Cameroon.

## Materials and Methods

### Study design and duration

This was a cross sectional study performed between October 2014 and September 2015.

### Study area

Buea (4°10′0″N 9°14′0″E) with an elevation of 870m (2,850ft) is located in the eastern slopes of Mount Cameroon. Buea is the capital of the Southwest Region of Cameroon. The population of Buea is estimated at 200,000 [30] and it is considered one of the fastest growing towns in Cameroon today with a mix cosmopolitan setting and a constellation of about 67 villages. The major ethnic group is the Bakweri (the indigenes). Because of its location at the foot of Mount Cameroon, the climate in Buea tends to be humid, with the neighbourhoods at higher elevations enjoying cooler temperatures while the lower neighbourhoods experience a hotter climate. Extended periods of rainfall, characterized by incessant drizzle, which can last for weeks, are common during the rainy season. The planning of Buea especially the villages is poorly done and several breeding sites for *Anopheles* mosquitoes can be seen around homes. Buea has two seasons; the dry season (between October and March), and the rainy season (between April and September). In Buea, human malaria can be described as mesoendemic in the dry season and hyperendemic in the rainy season, with peaks at the beginning and towards the end of the rainy season. The population of Buea experiences an estimated 3.93 infective bites/person/night [31]. *Plasmodium falciparum* accounts for up to 96% of malaria infections in this area [32]. In addition, Buea is also highly endemic for intestinal parasites including Ascaris lumbricoides, hookworm etc. [33,34]. School-age children are the most affected and as a result, mass deworming campaigns are organized annually [35] to curb down the burden.

### Study population

Febrile children (≤10 years) who came to consult in the outpatient department or emergency unit of the Regional Hospital of Buea were considered. The vital signs of the patients were taken and they were examined by the consulting physicians. Patients that were positive for malaria parasites by light microscopy were enrolled.

Excluded from the study were patients with a history of anti-malarial treatment within one week prior to admission. Patients were also excluded from the study based on evidence of other infectious disease, such as typhoid, gastroenteritis, meningitis, malnutrition, upper respiratory tract infections or any other identified cause of anaemia other than malaria.

### Ethical consideration

The study protocol was approved by the Institutional Review Board (IRB) of the Faculty of Health Sciences, University of Buea, Cameroon. Administrative clearance was obtained from the delegation of public health in the South West region of Cameroon. Written informed consent was obtained from the parents or guardians of the patients prior to their inclusion into the study.

### Specimen collection

Blood and stool specimen were collected from the participants. About 3ml of blood specimen was collected into test tubes containing EDTA anticoagulant following aseptic techniques. The parents or guardians were instructed to provide a teaspoon full of their child’s stool into a sterile leak-proof wide open neck stool container.

### Malaria microscopy and haemoglobin concentration determination

Thick and thin blood films were prepared and stained with 10% Giemsa and examined using methods previously described [36]. If parasites were observed, the density was determined by counting the number of parasites against 500 leucocytes. The parasite density was obtained by dividing the number of parasites counted by 500 and multiplying the result by the actual WBC count of the patient [37].

The haemoglobin concentration (Hb) was obtained from the complete blood count (CBC) results of the patient. The CBC was performed using the Mindray Auto haematology analyzer (BC-2800, Shenzhen Mindray Bio-Medical Electronics Co., Ltd).

### Determination of intestinal parasitic infection

The formol ether concentration technique (FECT) was used for the detection and quantification of intestinal parasites in stool samples from participants. Since hookworm eggs clear very rapidly, the FECT was performed within 30mins upon receipt of stool specimen following the proceedings described by Cheesbrough [38]. Briefly, about 1g of stool from each sample was emulsified in 4ml of 10% formalin in a conical tube with the aid of an applicator stick, to obtain a slightly cloudy suspension. A further 3ml of 10% formalin was added and thoroughly mixed by shaking (for approx. 30secs). The content of the tube was then strained through a surgical gauze into a beaker. The suspension was then transferred into another conical tube and 3ml of diethyl ether was added to it. The tube was closed with a rubber stopper and shaken vigorously for 1minute before centrifuging at 3000rpm for 5minutes. After spinning, the fatty plug (debris) was released from the sides of the tube with the aid of an applicator stick, and the supernatant was then poured away by quickly inverting the tube. The resulting sediment was then mixed by gently tapping the tubes with the fingers and the sediment was then transferred to a glass slide and covered with a cover glass. The whole area under the cover glass was then examined for ova, cysts, and larvae using the 10x and 40x objective of a light microscope. To assist in the identification of cysts, a small drop of iodine was added to the preparation under the cover glass. Helminths ova were separately counted for each species and recorded as previously described [39]. Helminths ova were counted until 100 ova were reached. Counts exceeding 100 were recorded as ‘100+’. A semi quantitative scheme was adopted for intestinal protozoa whereby samples were recorded as (i) ‘negative’ in cases where no cyst or trophozoite was observed in the entire sediment; (ii) ‘rare’ in cases where one to five cysts or trophozoites were observed per slide; (iii) ‘frequent’ in cases where one cyst or trophozoite was observed per field at 400x magnification; and (iv) ‘very frequent’ in cases where over one cysts or trophozoites were observed per field at 400x magnification.

### Categorization of malaria

Severe malaria (SM) was defined and categorized base on the WHO [40] criteria as follows; (1) severe anaemia (Hb<5g/dl) with no history of severe bleeding; (2) prostration, defined as the inability to sit upright or eat for a child who was able to do so; (3) respiratory distress, defined as difficulty in breathing with characteristic nasal flaring, subcostal recessions; (4) multiple convulsion reported within the preceding 24hrs plus one directly observed; (5) impaired consciousness, defined as a score ≤4 on the Blantyre Coma Scale [41]; (6) clinical jaundice; (7) circulatory collapse, defined as a systolic blood pressure <60 mm of Hg in children ≤5 years or < 80 mm of Hg in children > 5 years, in addition to observation of weak or absent of peripheral pulses or cold limbs; (8) abnormal bleeding; (9) pulmonary edema; and (10) frequent vomiting [42]. The term cerebral malaria was reserved for Blantyre coma score ≤2 corrected for no record of recent severe head trauma, neurological disease or any other cause of coma. And uncomplicated malaria (UM) defined by being fully conscious with haemoglobin ≥ 8g/dl and no signs of severity and/or evidence of vital organ dysfunction.

SM was further classified according to the degree of severity using the WHO scheme (S1 Table) into 3 groups with the first group being the most severe and associated with the highest mortality rate, the second group being moderately severe and the third being the least severe.

### Statistical analysis

Data collected were keyed into Excel spreadsheets and analysed using Stata version 12.1 (StataCorp LP). Statistical tests performed included the risk ratio, and the Pearson’s Chi square test for group comparison, student T test for the comparison of group means. Statistical significance was set at p < 0.05.

## Results

Out of the 637 children who were approached, 405 children met the inclusion criteria and were therefore enrolled. The ages of the children ranged between 1 week and 120 months (mean±SD = 41.5±33.5). Among the participants were 209 (51.6%) females and 196 (48.4%) males. Among the participants, 398 (98.3%) had single infection with *Plasmodium falciparum*, 5 (1.2%) had mixed infection with *P*. *falciparum* + *P*. *malariae*, and 2 (0.5%) had mixed infection with *P*. *falciparum* + *P*. *ovale*.

Severe malaria (SM) was observed in 44 (10.9%) of the 405 participants. All (100%) SM cases had single infection with *Plasmodium falciparum*. No significant association was observed between severe malaria and age (p = 0.377) nor was there any significant association between SM and gender (p = 0.387) (Table 1).

**Table 1:** Age and gender distribution of SM and UM in the study population.

Parameters	n	SM (n = 44)	UM (n = 361)	χ²	p-value
**Age category (Months)**					
< 60	290	34 (11.7)	256 (88.3)	0.78	0.377
60–120	115	10 (8.7)	105 (91.3)		
**Gender**					
Females	209	20 (9.6)	189 (90.4)	0.75	0.387
Males	196	24 (12.2)	172 (87.8)		

SM: severe malaria, UM: uncomplicated malaria

Stratified according to the degree of severity of SM, the distribution of the cases in decreasing order were: 26 (55.3%) for group 1; 17 (36.2%) for group 2; and 4 (8.5%) for group 3.

The main clinical presentation of SM in decreasing frequency were hyperpyrexia 30 (68.2%), severe malarial anaemia 27 (61.4%), multiple convulsion 23 (52.3%), circulatory collapse 19 (43.2%), respiratory distress 16 (36.4%), jaundice 15 (34.1%), protraction 11 (25%), hyperparasitaemia 7 (15.9%), impaired consciousness 7 (15.9%), hypoglycaemia 6 (13.6%), cerebral malaria (BCS≤2) 5 (11.4%), frequent vomiting 4 (9.1%), and coma 2 (4.6%).

**Fig 1:**
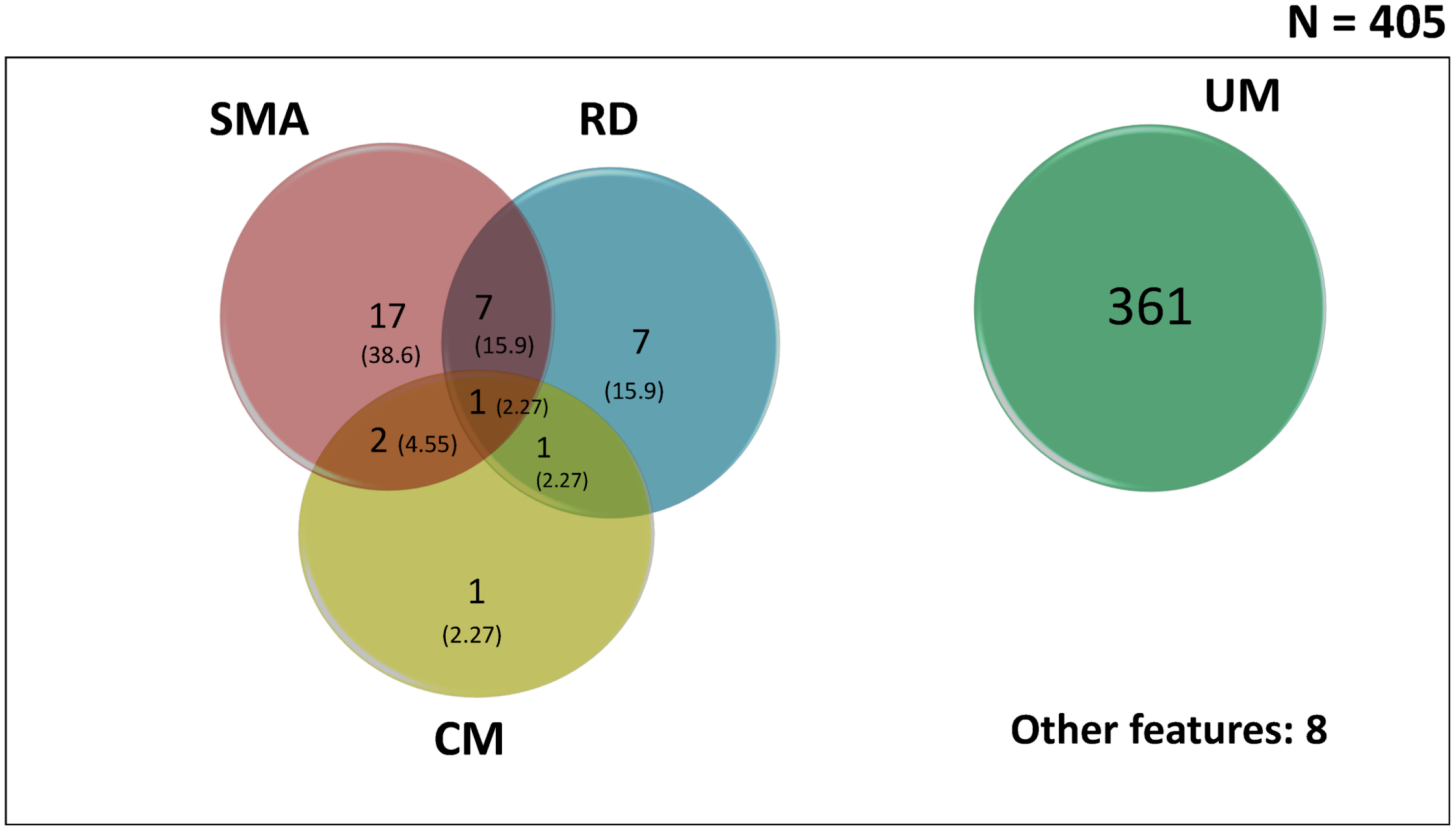
Venn diagram showing the overlap (proportions) of the major clinical subgroups of malaria in the study population. Proportions were obtained by dividing the cases by the total number of severe malaria (44). SMA: severe malarial anaemia; CM: cerebral malaria; RD: respiratory distress; UM: uncomplicated malaria.

The overlap of severe malaria anaemia with cerebral malaria and respiratory distress were observed in 4.6% (2/44) and 15.9% (7/44) participants respectively (Fig 1). One of the 44 (2.3%) participants with SM presented with all the three major clinical subgroups of malaria (severe malarial anaemia, cerebral malaria and respiratory distress).

Among the 405 participants, 47 were coinfected with intestinal parasites giving a prevalence of 11.6% (95% CI: 8.7–15.1). No significant association was observed between the prevalence of coinfection and gender (p = 0.484) (Table 2). On the contrary, there was a significant association between prevalence of coinfection and age (p = 0.003) (Table 2). The intestinal parasite isolated in decreasing order of prevalence were *Ascaris lumbricoides* 36 (69.2%), *Entamoeba spp*. 12 (23.1%), and Hookworm 4 (7.7%).

**Table 2:** Coinfection between intestinal parasites and malaria stratified according to age and gender.

Parameters	n	IPI present (%), n = 47	IPI absent (%), n = 358	χ²	p-value
**Age category (Months)**					
< 60	290	25 (8.6)	265 (91.4)	8.87	0.003
60–120	115	22 (19.1)	93 (80.9)		
**Gender**					
Females	209	22 (10.5)	187 (89.5)	0.49	0.484
Males	196	25 (12.8)	171 (87.2)		

IPI: intestinal parasitic infection

Among the coinfected children, 6/47 (12.8%) had SM meanwhile among non coinfected children, 38/358 had SM (10.6%). However no significant association was observed between the presence of coinfection and SM (χ² = 0.20, p = 0.656) (Table 3).

**Table 3:** Distribution of severe and uncomplicated malaria with IPI.

IPI	SM (%)	UM (%)	Total
**Present**	6 (12.8)	41 (87.2)	47
**Absent**	38 (10.6)	320 (89.4)	358
**Total**	**44**	**361**	**405**

SM: severe malaria, UM: uncomplicated malaria, IPI: intestinal parasitic infection

Stratified according to the degree of severity, the prevalence of coinfection was highest among cases of group 3 (mild SM) of the WHO classification (Table 4). A significant association was observed between the degree of severity of SM and the prevalence of coinfection (χ² = 7.21, p = 0.027).

**Table 4:** the distribution of IPI with respect to the degree of severity of SM.

Group	n	IPI present n (%)
**1**	26	1 (3.9)
**2**	17	3 (17.7)
**3**	4	2 (50.0)

IPI: intestinal parasitic infection

Species-specific analysis revealed no significant association between the different species of intestinal parasites and SM (χ² = 4.99, p = 0.172). Furthermore, the risk of developing SM was similar between all intestinal parasite species (Table 5).

**Table 5:** The association of the different species of intestinal parasite and severe malaria.

IPI	n	SM (%)	UM (%)	RR (95% CI)	p-value
**Only *A*. *lumbricoides***	31	2 (6.5)	29 (93.5)	1.00	
**Only Hookworm**	4	1 (25.0)	3 (75.0)	3.88 (0.45–33.70)	0.313
**Only *Entamoeba spp*.**	7	1 (14.3)	6 (85.7)	2.21 (0.23–21.14)	0.467
**A. *lumbricoides + Entamoeba spp*.**	5	2 (40.0)	3 (60.0)	6.20 (1.11–34.53)	0.084

IPI: intestinal parasitic infection, SM: severe malaria, UM: uncomplicated malaria, RR: risk ratio

The geometric mean parasite density (GMPD) in this study was 10332.7 (range: 65–160523). The GMPD was higher in children without IPI 10732.1 (range: 65–160523) compared to coinfected children 7290.6 (range: 171–82956). However this difference was not observed to be significant statistically (p = 0.185).

The average intensity (epg) of intestinal helminth was 28.2 (range: 2–134). The average intensity of intestinal helminths was higher in SM cases 36.8 (range: 8–105) compared to UM cases 26.9 (range: 2–134) (Table 6). However this difference was not observed to be significant (p = 0.272). The intensity of infection with *Entamoeba spp*. was generally rare across all groups (Table 6).

**Table 6:** The intensity of intestinal parasites stratified according to parasite species and type of malaria.

IPI	SM Mean ± SD	UM Mean ± SD	Total Mean ± SD
***A*. *lumbricoides***	42 ± 45.3	29.2 ± 33.5	30.7 ± 34.5
**Hookworm**	16 ± 0	4 ± 1	7 ± 6.1
***Entamoeba spp*.**^a^	rare	rare	rare
**Total**	36.8 ± 40.9	26.9 ± 32.7	28.2 ± 33.4

IPI: intestinal parasitic infection, SM: severe malaria, UM: uncomplicated malaria.

^a^rare: 1–5 cysts or trophozoites observed per slide; infection with intestinal protozoa was categorized as described by Utzinger et al. [39]

**Fig 2:**
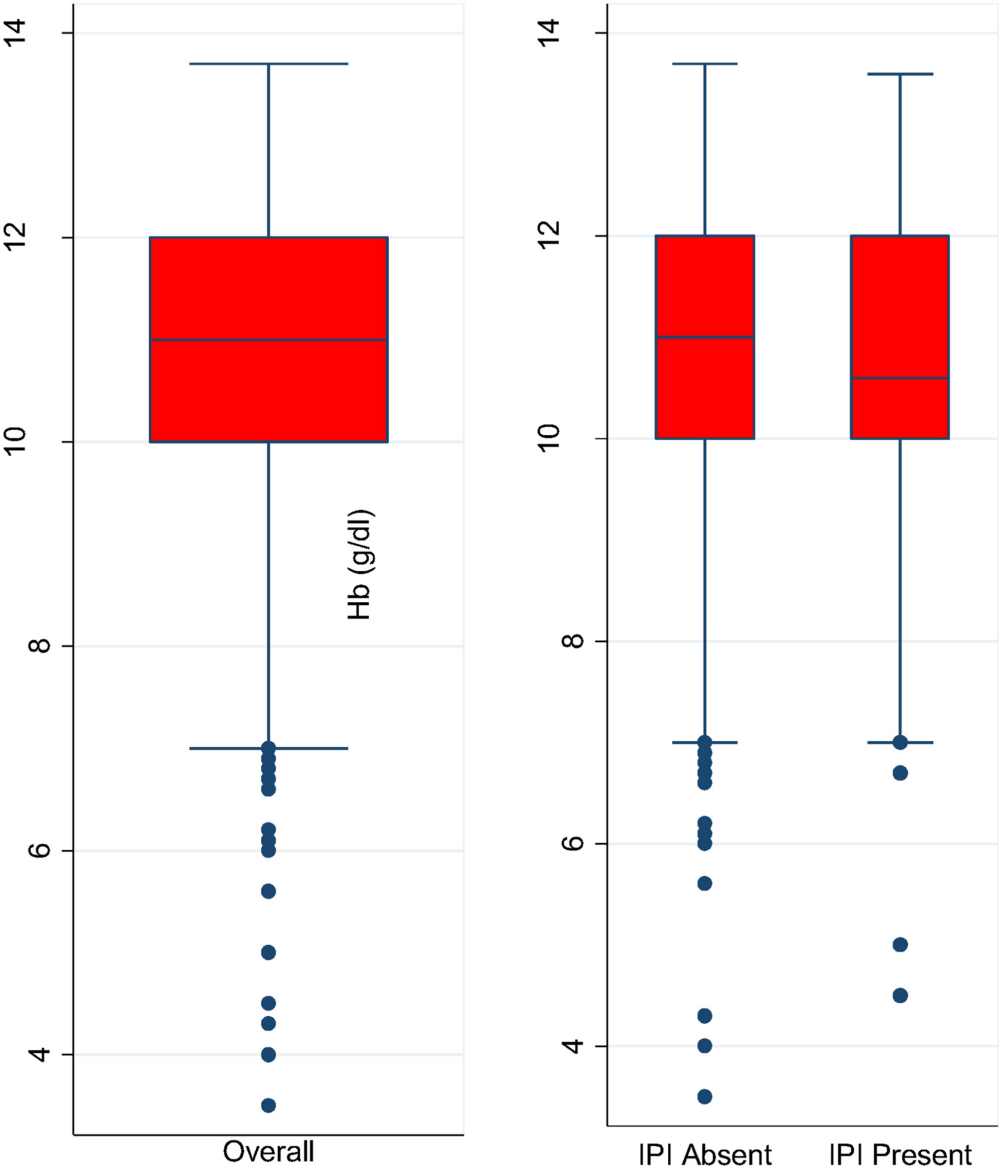
The distribution of mean Hb in the study population. No significant difference was observed in the mean Hb between children with IPI and those without (p = 0.205).

The mean Hb observed in this study was 10.6 (±1.8). The mean Hb was lower in children with IPI (10.4g/dl) compared to children without IPI (10.7g/dl) (Fig 2). However the difference in the mean Hb between children with IPI and those without was not observed to be significant statistically (p = 0.205).

The prevalence of anaemia were 44.4% (16/36), 50.0% (2/4), 57.1% (4/7) and 60.0% (3/5) in participants infected with *A*. *lumbricoides*, hookworm, *Entamoeba spp*. and mixed *A*. *lumbricoides/Entamoeba spp*. respectively. However no significant association was observed between anaemia and the infecting parasite species (χ^2^ = 0.18, p = 0.980).

## Discussion

In this study, 11.6% of the participants were coinfected with intestinal parasites. These findings are similar to that reported by Njunda et al. [28]. The prevalence is however lower compared to the 34.7% reported in communities around Dschang in the West region of Cameroon [43]. The difference in the prevalence reported in Dschang and this study could be attributed to differences in the study design; our study targeted children in an urban setting meanwhile theirs targeted mainly school-age children residing in rural settings. The prevalence of intestinal parasites observed in this study is lower compared to the 22.7% reported in Thailand [44], and the 34.2% reported in Ethiopia [45]. The lower prevalence of IPI observed in this study could be attributed to the regular deworming campaigns organized by the Cameroon’s Ministry of Public Health targeting mainly children in the study area. The prevalence of IPI was observed to be significantly higher in children in the age range 60–120 months, which is in line with studies performed elsewhere [28,43,46]. The higher prevalence of IPI in this age group could be attributed to the differences in the exposure as the children grow up, become more playful especially with soil. The most frequent intestinal parasite isolated was *Ascaris lumbricoides* (69.2%). Other studies have also shown that *Ascaris lumbricoides* is the most prevalent species of intestinal parasites isolated from children [25,46–49]. Hookworm infection is present in the study area but the prevalence observed is low (8.5%). The concentration method used in this study may have impacted the result of hookworm infection, therefore the real prevalence might have been underestimated.

In the current study, it was observed that 10.86% of the participants had severe malaria (SM) based on the case definition of severe malaria by the WHO [40]. The prevalence of SM in this study is similar to the prevalence reported by Tchokoleu et al. [50], but lower compared to the prevalence reported elsewhere in Cameroon [51,52], and in other countries [53–56]. The differences in the prevalence of SM observed in these studies and ours could be due to the ages of the target population—our study involved children 10 years and below meanwhile most of the studies performed elsewhere focused on children 5 years and below. The risk of SM has been shown to be higher in children below 5 years compared to older children due to the low immunity, which has been shown to increase with increasing age [40]. Furthermore, the level of endemicity of malaria could be another factor responsible for the differences in the prevalence observed in our study and the others; the endemicity of human malaria in the study area varies between mesoendemic in the dry season and hyperendemic in the rainy season. Although the prevalence of SM was observed to be higher in children below 5 years and in males in this study, no significant association was observed between SM and age, neither was there any association between SM and gender. The observation of higher prevalence of SM in males corroborate the studies performed elsewhere [55,56]. In this study, *P*. *falciparum* was the only parasite species observed to be associated with SM. *P*. *falciparum* is well recognised as the main cause of SM in endemic areas. Although there are reports of *P*. *vivax* also causing SM [57], *P*. *vivax* was not identified as a species causing malaria in this study. The main clinical features of SM in this study were hyperpyrexia, severe malaria anaemia and convulsion, which is similar to studies performed elsewhere [52,56].

In the current study, no significant difference was observed in the prevalence of severe malaria in children coinfected with intestinal parasites (12.8%) compared to malaria monoinfected children (10.6%). This is contrary to studies associating IPI and protection from severe malaria [18,20], as well as studies associating IPI and increased risk of SM [11–13]. Species-specific analysis also did not yield any significant association between the different intestinal parasite species and severe malaria (p = 0.281), which contradicts the study by Le Hesran et al. [12] in which infection with *Ascaris lumbricoides* was observed to be associated with increased attack of SM. This could be attributed to the differences in study design—our study was a cross sectional study involving children infected with malaria whereas theirs was a case-control study. Furthermore, no significant association was observed between the intensity of helminthic infection and SM in this study (p = 0.272). In this study, coinfection with malaria and intestinal parasites was significantly higher in group 3 (i.e. mild SM) of the WHO classification of SM (p = 0.027). The main phenotype of SM in this group is persistent vomiting; infection with malaria parasites or intestinal parasites are associated with gastrointestinal disturbances which may results in nausea and vomiting. Concomitant infection with both parasites may increase the progression of the gastrointestinal disturbances leading to increase in the frequency of vomiting. This observation should however be interpreted with caution as just a few (6) cases of IPI was observed among participants with SM. Larger studies will therefore be required to confirmed this association.

In the present study, the geometric mean parasite density was higher in malaria monoinfected children (10732.1 parasites/ul) compared to children coinfected with intestinal parasites (7290.6 parasites/ul). The interaction between intestinal parasites and malaria is not well understood. IPI especially helminths have been shown to have an immunomodulatory effect by inducing a Th2 host response. The host response will depend on the stage and intensity of helminth infection [57,58]. Clearly, the host response may also depend on the specific type of helminthic infection and the age of the host. However in this study, no significant difference was observed in the mean parasite density between children with IPI and those without IPI, which contradicts the study by Brutus et al. [16] in which treatment of *Ascaris lumbricoides* was observed to be associated with a 2-fold increase in malaria parasitaemia (suggesting a protective effect). The failure to observe any significant difference in the mean malaria parasite density between children with IPI and those without could be attributed to the study design. The cross sectional design of the present study, cannot differentiate between present and past infection; the Th2 polarization of immune responses to intestinal helminths due to previous exposure may still be maintained through certain period although the infection itself has been cleared at present.

The mean Hb observed in this study was low in children coinfected with IPI compared to children without IPI. IPI, especially infection with hookworm and *Trichuris trichiura*, cause anaemia by increasing blood and iron loss in the intestinal tract. However the difference in the mean Hb between IPI coinfected children and children without IPI was not observed to be significant (p = 0.205), which is contrary to the study by Nacher et al. [10]. Moreover, species specific analysis revealed no significant association between the infecting intestinal parasite species and anaemia. The failure to observe any significant difference in the mean Hb between children coinfected with IPI and those without could be attributed to the overall high prevalence of anaemia in this study as illustrated by the mean Hb 10.6(±1.8) for which there are many other causes including malnutrition, which is very rampant in and around communities in Buea.

The finding of no significant association between IPI and the clinical outcome of malaria in this study could be attributed to the low prevalence of IPI in the study area in addition to the other factors highlighted above. Studies involving larger number of cases with IPI are therefore required.

In conclusion, 11.6% of the participants were coinfected with intestinal parasites. The rate of severe malaria attack observed in the study was 10.9%. Severe malaria was not observed to be associated with age or gender. The prevalence of IPI was observed to be significantly higher in children with mild severe malaria. The main clinical presentations of severe malaria were hyperpyrexia, severe malarial anaemia and multiple convulsion. IPI was not observed to influence the severity of malaria, the malaria parasite density and the haemoglobin concentration in children in the study area, which is contrary to many studies published in the scientific literature. As it may appear, the clinical outcome of malaria in children coinfected with intestinal parasites may depend on the geographical setting.

## Supporting Information

S1 Table Bedside clinical classification of severe malaria in children in a high transmission area [42]. (DOCX)
