# Supplementary material for: The Effect of Intestinal Parasitic Infection on the Clinical Outcome of Malaria in Coinfected Children in Cameroon
Source: PLoS Negl Trop Dis. 2016 Apr 29;10(4):e0004673. doi: 10.1371/journal.pntd.0004673 (PMC4851403; doi:10.1371/journal.pntd.0004673)
Supplement: S1 Table — (DOCX) [file pntd.0004673.s001.docx]

| Group 1 | Prostrate children (prostration is the inability to sit upright in a child normally able to do so or to drink in the case of children too young to sit). Three subgroups of increasing severity should be distinguished:   - Prostrate but fully conscious - Prostrate with impaired consciousness but not in deep coma - Coma (the inability to localise a painful stimulus)   Respiratory distress (acidotic breathing):  Mild – sustained nasal flaring and/or mild intercostal indrawing (recession)  Severe – the presence of either marked indrawing (recession) of the bony structure of the lower chest wall or deep  (acidotic) breathing  Shock compensated or decompensated (see definition above) |
| --- | --- |
| Group 2 | Children who, although able to be treated with oral antimalarials, require supervised management because of the risk of clinical deterioration but who show none of the features of group 1 (above)*. These include children with any of the following:   - Haemoglobin <5 g/dl or haematocrit < 15% - 2 or more convulsions within a 24-h period - Haemoglobinuria (blackwater) - Jaundice - parasitaemia over 10% |
| Group 3 | Children who require parenteral treatment because of persistent vomiting but who lack any specific clinical or laboratory features of groups 1 or 2 (above) |
